# Evaluation des Projekts zur Einführung von Laienreanimation an Schulen in Nordrhein-Westfalen

**DOI:** 10.1007/s00101-020-00889-1

**Published:** 2020-11-26

**Authors:** Marc Felzen, Hanna Schröder, Stefan K. Beckers, Bernd W. Böttiger, Nadine Rott, Ruth Koch-Schultze, Sabine Wingen, Andreas Meißner, Iris Santowski, Olaf Picker, Niels Rahe-Meyer, Rico Dumcke, Claas Wegner, Hugo van Aken, Antje Gottschalk, Oliver Weber, Rolf Rossaint

**Affiliations:** 1 Klinik für Anästhesiologie, Medizinische Fakultät, RWTH Aachen University, Uniklinik RWTH Aachen, Pauwelsstr. 30, 52074 Aachen, Deutschland; 2 Klinik für Anästhesiologie und Operative Intensivmedizin, Universitätsklinikum Köln, Köln, Deutschland; 3 Klinik für Anästhesiologie, Klinikum Stadt Soest, Soest, Deutschland; 4 Klinik für Anästhesiologie, Universitätsklinikum Düsseldorf, Düsseldorf, Deutschland; 5 Klinik für Anästhesiologie und operative Intensivmedizin, Franziskus Hospital, Bielefeld, Deutschland; 6 Fakultät für Biologie, Biologiedidaktik, Universität Bielefeld, Bielefeld, Deutschland; 7 Klinik für Anästhesiologie, operative Intensivmedizin und Schmerztherapie, Universitätsklinikum Münster, Münster, Deutschland; 8 Zentrum für Anästhesiologie, Intensivmedizin und Schmerztherapie, Klinikum Vest, Recklinghausen, Deutschland

**Keywords:** Wiederbelebung in Schulen, Ein Leben retten, Herzstillstand, Erste Hilfe, Ministerium für Schule und Bildung, Resuscitation in schools, Safe a live, Cardiac arrest, First aid, Ministry for schols and education

## Abstract

**Hintergrund:**

Wiederbelebungsunterricht ist nicht in allen Schulen Deutschlands verpflichtend, dieser beschränkt sich trotz niedriger Laienreanimationsrate aktuell auf einzelne, z. T verpflichtende Projekte in unterschiedlichen Bundesländern. Aus diesem Grunde hat das Ministerium für Schule und Bildung Nordrhein-Westfalen per Runderlass im März 2017 das Projekt „Laienreanimation an Schulen in NRW“ initiiert.

**Fragestellung:**

Ziel dieser Arbeit ist die Evaluation dieses Projekts.

**Material und Methoden:**

Alle weiterführenden Schulen in Nordrhein-Westfalen wurden zur Teilnahme am Projekt eingeladen. Aus jedem Regierungsbezirk nahmen ärztliche Partner teil, welche Wiederbelebungstrainings mit bereits bestehenden Konzepten zum Lehrer- oder Schülertraining durchführten. Nach einer 3‑jährigen Laufzeit erfolgte die Evaluation anhand von standardisierten Fragebögen für Schuldezernenten, Lehrer und Schüler.

**Ergebnisse:**

Insgesamt konnten durch das Projekt mehr als 40.000 Schüler aus 249 Schulen in NRW mit 6 unterschiedlichen Konzepten in Wiederbelebung trainiert werden. Fragen bezüglich Wiederbelebungsmaßnahmen konnten durch 85 % der Schüler richtig beantwortet werden. Die Schüler fühlen sich insgesamt sicher in Wiederbelebungsmaßnahmen. Der Investitionsbedarf für alle Schulen liegt bei einmalig zwischen 4 und 6,5 Mio. € sowie rund 340.000 € in jedem Haushaltsjahr.

**Diskussion:**

Eine gesetzliche Verpflichtung und Finanzierung von Wiederbelebungstrainings sind unerlässlich für eine flächendeckende Durchführung. Alle durchgeführten Konzepte sind effektiv, dementsprechend kann jede Schule ein auf ihre Bedürfnisse abgestimmtes Konzept auswählen und dieses bestenfalls gestuft anwenden. Die Schulung von Lehrern sollte gezielt auf Wiederbelebung ausgerichtet sein.

**Zusatzmaterial online:**

Die Online-Version dieses Beitrags (10.1007/s00101-020-00889-1) enthält eine Kostenaufstellung der Bereitstellung und Wartung von Wiederbelebungspuppen.

Beitrag und Zusatzmaterial stehen Ihnen auf www.springermedizin.de zur Verfügung. Bitte geben Sie dort den Beitragstitel in die Suche ein, das Zusatzmaterial finden Sie beim Beitrag unter „Ergänzende Inhalte“.

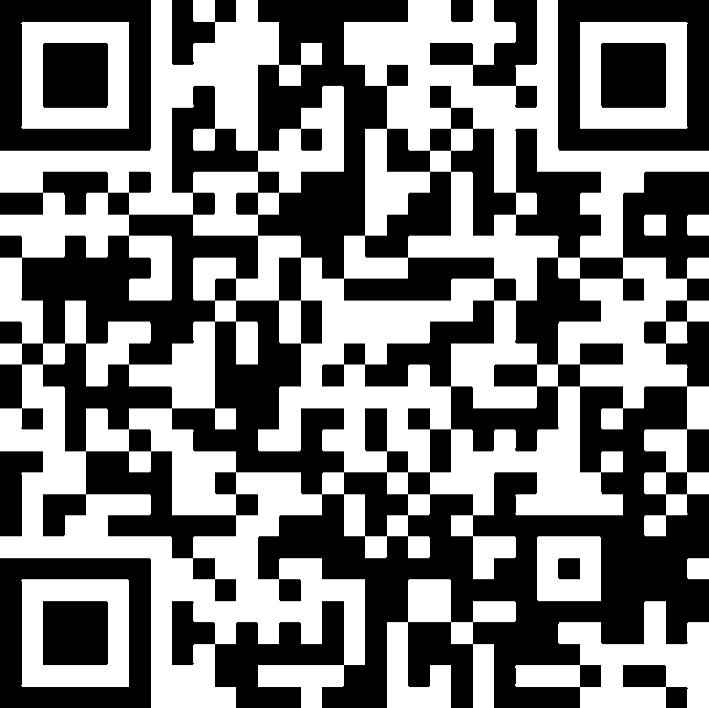

## Abstract (en)

**Background:**

Teaching of resuscitation measures is not mandatory in all schools in Germany. It is currently limited to individual, partly mandatory projects despite a low bystander resuscitation rate. For this reason, the Ministry for Schools and Education of North Rhine-Westphalia initiated the project “Bystander resuscitation at schools in NRW” in March 2017.

**Objective:**

The aim of this work was to evaluate this project.

**Material and methods:**

All secondary schools in North Rhine-Westphalia were invited to participate in the project. Medical partners from each administrative district took part, who carried out resuscitation training with existing concepts for teacher or student training. After a 3-year period, the evaluation was carried out using standardized questionnaires for school headmasters, teachers and students.

**Results:**

In total, more than 40,000 pupils from 249 schools in NRW could be trained in resuscitation within the project with 6 different concepts. Of the students 85% answered the questions regarding resuscitation correctly and overall felt safe in resuscitation measures. The one-off investment requirement for all schools is roughly 4–6.5 million € and around 340,000 € in each budget year.

**Conclusion:**

A legal constitution and funding are necessary for a nationwide introduction of resuscitation in schools. All established concepts are effective, therefore each school can use them exactly according to their needs, optimally in a stepped form. Training for teachers should focus on resuscitation.

## Hintergrund

Wiederbelebungsunterricht ist an Schulen in Nordrhein-Westfalen (NRW) und auch deutschlandweit bisher nicht etabliert; dieser beschränkt sich auf einzelne Projekte, welche allerdings allesamt erfolgreich sind [8, 10, 14]. Dabei hat der Schulausschuss der Kultusministerkonferenz bereits im Juni 2014 die Einführung von Modulen zum Thema Wiederbelebung befürwortet und den Ländern empfohlen, Lehrkräfte entsprechend ausbilden zu lassen. Darüber hinaus soll laut Koalitionsvertrag NRW zwischen der Christlich Demokratischen Union Deutschlands (CDU) und der Freien Demokratischen Partei (FDP) 2017–2022 durch die Unterrichtung an allen Schulen in Nordrhein-Westfalen die Bereitschaft zu Erster Hilfe und Wiederbelebung von Anfang an gefördert werden. Hintergrund ist eine niedrige Laienreanimationsrate in Deutschland, welche zu geringen Überlebenschancen eines Herzstillstands führt. Ursächlich dafür ist das Absterben von Hirnzellen bereits 5 min nach dem Herzstillstand ohne Wiederbelebungsmaßnahmen, also meistens noch vor Eintreffen des Rettungsdienstes. Durch unterschiedliche Bestrebungen ist die Laienreanimationsrate in den letzten Jahren bereits von 17 % auf 39 % [35] angestiegen, Deutschland liegt jedoch im europäischen Vergleich immer noch deutlich unterhalb des Durchschnitts von 58 % [16]. So konnte die Laienreanimationsrate in Dänemark durch die landesweite Einführung von Pflichtunterricht zum Thema Wiederbelebung von 20 % im Jahre 2001 auf mehr als 50 % im Jahre 2012 gesteigert werden [24]. Während Wiederbelebungsunterricht in Belgien, Dänemark, Frankreich, Italien und Portugal im Rahmen der globalen, von der Weltgesundheitsorganisation unterstützten, Initiative „Kids save lives“ [9, 11, 12] gesetzlich verankert ist, wird er in den meisten Ländern bisher lediglich empfohlen. In Deutschland existieren bisher nur für die Bundesländer Baden-Württemberg über die im September 2015 gestartete Initiative „Löwen retten Leben“, Bayern über eine Bekanntmachung des Bayerischen Staatsministeriums für Unterricht und Kultus und Mecklenburg-Vorpommern über das Projekt „Retten macht Schule“ bundeslandweite Konzepte [1, 3, 22, 31], welche die Ausbildung von Lehrern für das Training von Schülern vorsehen. Diesbezüglich existieren bereits Ausbildungscurricula für Lehrer [11, 29] und Schüler seitens des Deutschen Rates für Wiederbelebung (GRC), deren Effektivität bereits gezeigt werden konnte [6, 8, 10, 14, 27].

Zur Erhöhung der Laienreanimationsrate in NRW hat das Ministerium für Schule und Weiterbildung NRW per Runderlass vom 20.03.2017 [2] zum ersten August 2017 das Projekt „Laienreanimation an Schulen in NRW“ initiiert, welches von der Deutschen Gesellschaft für Anästhesiologie, dem Berufsverband Deutscher Anästhesisten sowie dem GRC unterstützt wird. Die Laufzeit des Projekts betrug 3 Jahre. Ziel war es, möglichst viele an Schule beteiligte Personen, insbesondere Schülerinnen und Schüler, aber auch Lehr- und Fachkräfte sowie Eltern mit Wiederbelebungsmaßnahmen vertraut zu machen sowie für die regelmäßige Durchführung dieser zu gewinnen. Ziel dieser Arbeit ist die Evaluation dieses Projekts, verbunden mit der Fragestellung, wie Wiederbelebungsunterricht in Schulen flächendeckend eingeführt werden kann.

## Methode

Unter Federführung des Ministeriums für Schule und Bildung (MSB) NRW wurde eine Steuergruppe gebildet, bestehend aus der Referatsleitung Gesundheit des MSB, Schuldezernenten der 5 NRW-Regierungsbezirke, ärztlichen Partnern aus allen 5 NRW-Regierungsbezirken (Tab. 1) sowie dem Referatsleiter Rettungswesen des Ministeriums für Arbeit, Gesundheit und Soziales (MAGS) NRW und einem Vertreter des Betriebskrankenkassen Landesverband Nordwest (BKK LV Nordwest).

**Table 1:** 

Regierungsbezirk	Arnsberg	Detmold	Köln		Düsseldorf	Münster
Koordinierende Klinik	Klinikum Stadt Soest	Franziskus Hospital Bielefeld	Uniklinik Aachen	Uniklinik Köln	Uniklinik Düsseldorf	Uniklinik Münster
Gesamtdauer [min]	90	90	45	90	90	90
Dauer, Theorie [min]	45	45	20	45	45	45
Dauer, Praxis [min]	45	45	25	45	45	45
Dozenten	Lehrer	Lehrer	Notärzte	Lehrer	Lehrer	Lehrer
Anzahl, Schüler/ Durchgang	1 Klasse	1 Jahrgang	1 Jahrgang	1 Klasse	1 Klasse	1 Klasse
Training	Herzdruckmassage	Herzdruckmassage (ggf. + Beatmung + AED)	Herzdruckmassage	Herzdruckmassage	Herzdruckmassage	Herzdruckmassage
Wiederholung	2‑jährlich	Jährlich	Jährlich	Jährlich	Jährlich	Jährlich

Zunächst wurden alle weiterführenden Schulen in NRW schriftlich zur Teilnahme am Projekt eingeladen. Das MAGS und der BKK LV Nordwest stellten Spendengelder für die Anschaffung von Wiederbelebungspuppen zur Verfügung. Nach Durchführung einer Ausschreibung wurde das Modell „Practi-Man“ (Fa. Vimetecsa, Alicante, Spanien) beschafft und in Klassensätzen an alle teilnehmenden Schulen ausgeliefert.

Die Durchführung des Projektes erfolgte mithilfe von durch die ärztlichen Partner in den einzelnen Regierungsbezirken bereits etablierten unterschiedlichen Schulungskonzepten. Diese sahen entweder eine 2‑ bis 4‑stündige Schulung von Lehrern in Anlehnung an den modularen Lehrerausbildungskurs des GRC [13] zum Trainieren der Schüler oder aber ein direktes Training von Schülern vor. Grundlage bildete die Trias „Prüfen – Rufen – Drücken“ der Aktion „Ein Leben retten“ [4], standortabhängig erweitert um Beatmung und automatischen externen Defibrillator (Tab. 1). Sämtliches Unterrichtsmaterial wurde den Lehrern zur Verfügung gestellt. Weder Lehrer noch Schüler erhielten im Rahmen des Projekts einen zertifizierten Erste-Hilfe-Kurs; Fokus der Schulungen war die Wiederbelebung.

**Table 2:** 

Regierungsbezirk	Arnsberg	Detmold	Köln		Düsseldorf	Münster
			Aachen	Köln		
*Zahlen*						
Schulen	65	67	23	26	26	42
Jahrgang	7 + 8	7–12	7–12	7–12	7–12	7–12
Schüler	9000	5029	10634	8249	1952	>6000
Lehrer	437	194	11	223	39	110
Studenten	0	72	17	0	0	0
*Dozenten*						
Lehrer	✔	✔	☐	✔	✔	✔
Studenten	✔^a^	✔^b^	☐	☐	☐	☐
Ärzte	☐	☐	✔	☐	☐	☐
Hilfsorganisation	☐	☐	☐	☐	☐	☐

^a^Universität Witten-Herdecke

^b^Universität Bielefeld

Die Evaluation erfolgte anhand von standardisierten Fragebögen für die Schuldezernenten, Lehrer und Schüler (Anlage 1–3), wobei die Lehrer- und Schülerbefragung nur stichprobenartig in allen Regierungsbezirken erfolgte.

Signifikanztestungen erfolgten mit dem Exakten Test nach Fisher auf einem Signifikanzniveau von 5 %.

## Ergebnisse

Insgesamt konnten zwischen August 2017 und August 2020 durch das Projekt mit 6 bestehenden Konzepten mehr als 40.000 Schüler in NRW in Wiederbelebung trainiert werden. Es nahmen 249 (9,2 %) der 2708 weiterführenden Schulen in NRW am Projekt teil. Darüber hinaus wurden 1103 Multiplikatoren (1014 Lehrer, 89 Studenten) ausgebildet.

Mithilfe von Spendengeldern konnten nach Ausschreibung 2974 Wiederbelebungspuppen Practi-Man der Fa. Vimetecsa sowie für den Regierungsbezirk Münster 600 Mini Anne (Fa. Laerdal, Puchheim, Deutschland) beschafft und an die Schulen verteilt werden. Der Practi-Man zeigte sich robust auch nach Durchführung von mehreren Wiederbelebungstrainings. Einzig die individuelle Nachbestellung von Beatmungszubehör durch die Schulen gestaltete sich schwierig. Die Mini Anne wurde den Schülern nach dem Training geschenkt und dementsprechend nicht wiederverwendet.

### Lehrerbefragung

Es liegen Ergebnisse von 71 Lehrkräften vor. Die Lehrkräfte wünschen sich eine verpflichtende Verankerung des Wiederbelebungsunterrichts im Lehrplan sowie einen Start bereits in der Grundschule, verbunden mit einem fertigen Konzept, Praxishilfen und Leitfäden. Weiterhin sollen Schulsanitäter zur Unterstützung der Wiederbelebungstrainings ausgebildet werden. Darüber hinaus sind die Lehrer der Meinung, dass Laienreanimation nicht privaten Anbietern überlassen werden kann, da die Schüler trotz eines solchen Kurses nicht handlungsfähig sind.

Aus Tab. 3 geht hervor, dass die Lehrer die Relevanz des Themas durchschnittlich als hoch einschätzen und dementsprechend motiviert sind.

**Table 3:** 

Frage		Arnsberg	Detmold	Köln		Düsseldorf	Münster	Gesamt
				Aachen	Köln			
Anzahl		19 (100 %)	19 (100 %)	12 (100 %)	3 (100 %)	12 (100 %)	8 (100 %)	73 (100 %)
Relevanz	Sehr hoch	6 (31,6 %)	6 (31,6 %)	1 (8,3 %)	2 (66,7 %)	7 (58,3 %)	4 (50,0 %)	26 (41,1 %)
	Eher hoch	12 (63,2 %)	6 (31,6 %)	4 (33,3 %)	0 (0,0 %)	3 (25,0 %)	3 (37,5 %)	28 (31,8 %)
	Eher gering	1 (5,3 %)	4 (21,1 %)	3 (25,0 %)	1 (33,3 %)	1 (8,3 %)	1 (12,5 %)	11 (17,6 %)
	Sehr gering	0 (0,0 %)	2 (10,5 %)	4 (33,3 %)	0 (0,0 %)	3 (25,0 %)	0 (0,0 %)	9 (11,5 %)
Motivation	Sehr hoch	11 (57,9 %)	5 (26,3 %)	4 (33,3 %)	2 (66,7 %)	4 (33,3 %)	4 (50,0 %)	30 (44,6 %)
	Eher hoch	6 (31,6 %)	8 (42,1 %)	3 (25,0 %)	0 (0,0 %)	6 (50,0 %)	3 (37,5 %)	26 (31,0 %)
	Eher gering	2 (10,5 %)	6 (31,6 %)	5 (41,7 %)	1 (33,3 %)	1 (8,3 %)	0 (0,0 %)	15 (20,9 %)
	Sehr gering	0 (0,0 %)	0 (0,0 %)	0 (0,0 %)	0 (0,0 %)	1 (8,3 %)	0 (0,0 %)	1 (1,4 %)
Bereits Schüler trainiert	Ja	15 (78,9 %)	16 (84,2 %)	12 (100,0 %)	3 (100,0 %)	8 (66,7 %)	7 (87,5 %)	61 (86,2 %)
	Nein	4 (21,1 %)	3 (15,8 %)	1 (8,3 %)	0 (0,0 %)	4 (33,3 %)	0 (0,0 %)	12 (13,1 %)
Mit Lehrplan verbunden	Ja	9 (47,4 %)	11 (57,9 %)	5 (41,7 %)	1 (33,3 %)	4 (33,3 %)	1 (12,5 %)	31 (37,7 %)
	Nein	10 (52,6 %)	8 (42,1 %)	7 (58,3 %)	2 (66,7 %)	8 (66,7 %)	6 (75,0 %)	41 (60,2 %)
Durchführung^a^	Schulstunden	10 (52,6 %)	9 (47,4 %)	1 (8,3 %)	–	12 (100,0 %)	3 (37,5 %)	35 (41,0 %)
	Aktionstage	8 (42,1 %)	7 (36,8 %)	3 (25,0 %)	–	1 (8,3 %)	5 (62,5 %)	24 (29,1 %)
	Projektwoche	0 (0,0 %)	5 (26,3 %)	0 (0,0 %)	–	2 (16,7 %)	1 (12,5 %)	8 (9,2 %)
Ausreichend vorbereitet zu unterrichten	Ja	13 (68,4 %)	10 (52,6 %)	4 (33,3 %)	2 (66,7 %)	9 (75,0 %)	5 (62,5 %)	43 (59,8 %)
	Nein	6 (31,6 %)	9 (47,4 %)	8 (66,7 %)	1 (33,3 %)	3 (25,0 %)	1 (12,5 %)	28 (36,1 %)
Zufriedenheit ärztliche Partner	Sehr zufrieden	7 (36,8 %)	9 (47,4 %)	8 (66,7 %)	–	7 (58,3 %)	3 (37,5 %)	34 (41,1 %)
	Eher zufrieden	4 (21,1 %)	7 (36,8 %)	0 (0,0 %)	–	5 (41,7 %)	2 (25,0 %)	18 (20,8 %)
	Eher nicht zufrieden	0 (0,0 %)	0 (0,0 %)	0 (0,0 %)	–	0 (0,0 %)	0 (0,0 %)	0 (0,0 %)
	Gar nicht zufrieden	1 (5,3 %)	0 (0,0 %)	0 (0,0 %)	–	0 (0,0 %)	1 (12,5 %)	2 (3,0 %)
Unterrichtsmaterial^a^	Arbeitsblätter	14 (73,7 %)	16 (84,2 %)	4 (33,3 %)	3 (100,0 %)	7 (58,3 %)	7 (87,5 %)	51 (72,8 %)
	Flyer	9 (47,4 %)	15 (78,9 %)	0 (0,0 %)	0 (0,0 %)	1 (8,3 %)	5 (62,5 %)	30 (32,9 %)
	Informationsbroschüren	9 (47,4 %)	13 (68,4 %)	4 (33,3 %)	1 (33,3 %)	4 (33,3 %)	5 (62,5 %)	36 (46,4 %)
	Funktionsmodelle	8 (42,1 %)	13 (68,4 %)	4 (33,3 %)	1 (33,3 %)	4 (33,3 %)	5 (62,5 %)	35 (45,5 %)
	Sonstiges	0 (0,0 %)	0 (0,0 %)	1 (8,3 %)	0 (0,0 %)	0 (0,0 %)	0 (0,0 %)	1 (1,4 %)
Leitfäden gewünscht	Ja	12 (63,2 %)	15 (78,9 %)	10 (83,3 %)	3 (100,0 %)	8 (66,7 %)	7 (87,5 %)	55 (79,9 %)
	Nein	7 (36,8 %)	3 (15,8 %)	2 (16,7 %)	0 (0,0 %)	4 (33,3 %)	0 (0,0 %)	16 (17,1 %)
Verbindliches Konzept	Ja	9 (47,4 %)	7 (36,8 %)	3 (25,0 %)	2 (66,7 %)	1 (8,3 %)	1 (12,5 %)	23 (32,8 %)
	Nein	10 (52,6 %)	10 (52,6 %)	9 (75,0 %)	0 (0,0 %)	11 (91,7 %)	7 (87,5 %)	47 (59,9 %)

^a^Mehrere Antworten möglich

Die meisten Lehrer sind zufrieden mit den Schulungen der ärztlichen Partner und fühlen sich ausreichend vorbereitet, Schüler zu trainieren, und haben dies auch bereits vornehmlich in Schulstunden getan. Wiederbelebungstrainings sind jedoch größtenteils noch nicht im Lehrplan integriert. Auch fehlt es den Lehrern an einem verbindlichen Konzept. Darüber hinaus sind Informationsmaterial und ein Leitfaden gewünscht.

### Schülerbefragung

**Figure 1:**
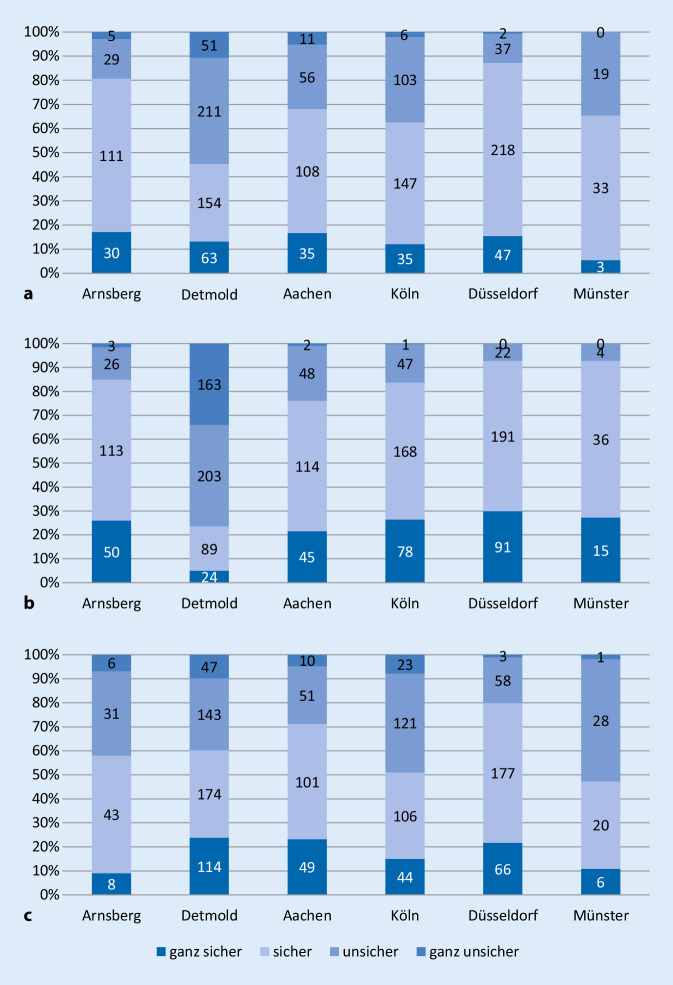

Insgesamt konnten 1657 Schülerfragebögen aller Schulformen ausgewertet werden. Aus Tab. 4 geht hervor, dass mindestens 85 % der Schüler aller Regierungsbezirke, welche den Fragebogen abgegeben haben, die Fragen bezüglich Erkennen des Kreislaufstillstands und der Durchführung von Wiederbelebungsmaßnahmen richtig beantwortet haben. In der Vergangenheit nahmen bereits 1140 (71,2 %) Schüler an einem Wiederbelebungstraining teil. Diese beantworteten die Fragen signifikant häufiger richtig (88,8 % vs. 84,1 %; *p* = 0,02). Insgesamt fühlen sich die Schüler sicher, einen Kreislaufstillstand zu erkennen und Wiederbelebungsmaßnahmen durchzuführen. Unsicherheiten gibt es v. a. im Regierungsbezirk Detmold beim Erkennen einer normalen Atmung. Im Regierungsbezirk Münster fühlen sich mehr als die Hälfte der Schüler unsicher bei der Durchführung der Herzdruckmassage (Abb. 1).

**Table 4:** 

Frage		Arnsberg	Detmold	Köln		Düsseldorf	Münster	Gesamt
				Aachen	Köln			
Anzahl, Schüler		305	479	213	294	311	55	1657
Anzahl, Schulen [% des Regierungsbezirks]		6 (2,0 %)	6 (1,3 %)	3 (1,4 %)	2 (0,7 %)	3 (1,0 %)	1 (1,8 %)	21 (1,3 %)
Durchschnittsalter		14	15	14	14	17	15	15
Alter von/bis		12–17	10–32	10–20	10–22	10–33	14–20	10–33
[*Alle folgenden Angaben in Relation zur Anzahl der Schüler*]								
Geschlecht	Weiblich	35,70 %	61,60 %	45,50 %	56,10 %	70,40 %	67,30 %	56,10 %
	Männlich	29,80 %	38,40 %	54,50 %	43,50 %	27,00 %	32,70 %	37,70 %
Vorher teilgenommen	Ja	53,80 %	72,20 %	64,80 %	86,70 %	60,50 %	89,10 %	71,20 %
	Nein	9,80 %	27,60 %	33,80 %	13,30 %	36,00 %	9,10 %	21,60 %
Vorgehen, regungslose Person	Richtig	85,60 %	90,00 %	73,20 %	87,10 %	92,60 %	90,90 %	86,60 %
	Falsch	13,80 %	9,80 %	26,30 %	12,60 %	7,10 %	9,10 %	13,10 %
Notrufnummer	Richtig	98,40 %	96,50 %	97,70 %	95,90 %	99,40 %	100,00 %	98,00 %
	Falsch	1,30 %	3,50 %	2,30 %	4,10 %	0,60 %	0,00 %	2,00 %
Wann Beginn der Herzdruckmassage	Richtig	84,30 %	87,70 %	82,60 %	85,70 %	89,70 %	83,60 %	85,60 %
	Falsch	15,40 %	12,10 %	16,90 %	12,20 %	10,30 %	12,70 %	13,30 %
Überprüfung der Atmung	Richtig	84,30 %	88,10 %	71,80 %	86,70 %	88,70 %	92,70 %	85,40 %
	Falsch	15,40 %	11,70 %	27,20 %	13,30 %	10,90 %	5,50 %	14,00 %
Funktion, Herz	Richtig	98,70 %	99,40 %	83,10 %	98,30 %	98,10 %	98,20 %	96,00 %
	Falsch	1,00 %	0,60 %	15,00 %	1,70 %	1,90 %	0,00 %	3,40 %
Was ist Herzdruckmassage	Richtig	86,20 %	86,60 %	68,50 %	90,80 %	88,70 %	90,90 %	85,30 %
	Falsch	13,40 %	13,40 %	28,60 %	8,80 %	10,00 %	9,10 %	13,90 %
Handposition	Richtig	92,50 %	85,80 %	89,70 %	87,80 %	97,70 %	85,50 %	89,80 %
	Falsch	7,20 %	14,20 %	8,90 %	11,90 %	1,90 %	12,70 %	9,50 %
Druckfrequenz	Richtig	81,30 %	52,60 %	88,30 %	66,70 %	92,60 %	81,80 %	77,20 %
	Falsch	18,40 %	47,40 %	10,30 %	32,70 %	7,10 %	18,20 %	22,30 %
Dauer, Überleben der Hirnzellen	Richtig	87,50 %	77,20 %	92,50 %	86,40 %	94,50 %	87,30 %	87,60 %
	Falsch	11,50 %	22,30 %	5,60 %	13,60 %	4,80 %	12,70 %	11,80 %

### Dezernentenbefragung

Die Befragung der Schuldezernenten aus den 5 Regierungsbezirken ergab das Erfordernis, Wiederbelebungsmaßnahmen verpflichtend in den Lehrplan zu integrieren. Um die Schulen bis dahin zur Teilnahme am Projekt zu motivieren, sollte die Wichtigkeit des Themas durch die ärztlichen Partner regelmäßig auf Schulleiterdienstbesprechungen der weiterführenden Schulen dargelegt werden. Die notwendige Schulung von Lehrkräften sollte im Rahmen von kurzen, regelmäßigen Lehrerfortbildungen durchgeführt werden, was z. B. mithilfe von kooperierenden Kliniken in der Nähe erfolgen kann. Die Kopplung mit einem Erste-Hilfe-Kurs ist aufgrund des nur geringen Anteils an Wiederbelebung sowohl zeitlich als auch inhaltlich nicht sinnvoll.

Die im Rahmen des Projekts durchgeführten Konzepte müssen auf einer Informationsplattform zur Verfügung gestellt werden. Hierfür bieten sich z. B. das Bildungsportal NRW (www.schulministerium.nrw.de) und die Seite des Landesprogramms Bildung und Gesundheit an (www.bug-nrw.de) an. Weiterhin muss die Versorgung mit Übungsmaterial hinsichtlich Neubeschaffung und Nachbestückung mit Verbrauchsmaterial für alle interessierten Schulen sichergestellt sein.

### Kosten

Für die Ausstattung aller Schulen mit Wiederbelebungspuppen liegt der Einmalinvestitionsbedarf in NRW zwischen 4 und 6,5 Mio. €. Es ist davon auszugehen, dass die Puppen mindestens 5 Jahre eingesetzt werden können. Zusätzlich benötigt man unabhängig vom durchgeführten Trainingskonzept (Lehrer- oder direktes Schülertraining) etwa 2 €/Schüler und Training in den ersten 2 bis 3 Jahren. Dies bedeutet, dass man in NRW bei 170.000 Schülern der 8. Klasse pro Schuljahr rund 340.000 € in jedem Haushaltsjahr einplanen muss (Zusatzmaterial online, s. Hinweisbox am Anfang des Beitrags).

## Diskussion

Im Rahmen dieser Arbeit wurde die Einführung von Wiederbelebungsunterricht im Rahmen des Projekts „Laienreanimation an Schulen in NRW“ evaluiert. Alle im Rahmen dieses Projekts durchgeführten Konzepte zeigten sich vergleichbar effektiv wie in zahlreichen vorherigen Projekten.

Die Teilnahme von 9,2 % aller weiterführenden Schulen in NRW stellt einen guten Anfang dar, muss jedoch zwingend ausgebaut werden. Um eine flächendeckende Durchführung von Wiederbelebungstrainings in Schulen von NRW zu erreichen, ist eine gesetzlich geregelte Verpflichtung erforderlich, wie bereits in 5 europäischen Ländern erfolgt, bestenfalls bereits ab der Grundschule [7, 15, 20, 23]. Bereits 2 Schulstunden/Schuljahr reichen aus [8], um eine nachhaltige Verbesserung der Kenntnisse in Wiederbelebung zu erreichen. Bis zur gesetzlichen Verpflichtung sollten bereits jetzt vorliegende Konzepte zur Durchführung von Lehrer- und Schülerausbildung genutzt und durch das Ministerium für Schule und Bildung vorangetrieben werden. Auch ist das kontinuierliche Aufzeigen der Wichtigkeit des Themas in Leitungsbesprechungen erforderlich.

Sowohl die Durchführung durch Lehrer als auch durch Ärzte zeigte sich effektiv [8, 19], ein kompaktes Training aller Schüler einer Schule an einem Tag ist nur mit dem Aachener Konzept erfolgt. Hierfür war jedoch auch der größte Material- und Personaleinsatz erforderlich. Zur Durchführung aller anderen Konzepte sind regelmäßige, speziell auf die Wiederbelebung ausgerichtete Lehrerfortbildungen erforderlich, um Motivation und Sicherheit zur Durchführung der Wiederbelebungstrainings zu erreichen [11, 26]. Hier haben verschiedene Angebote unterschiedlicher ärztlicher Partner genauso wie vorherige Untersuchungen [23] gezeigt, dass eine erfolgreiche Umsetzung mit verschiedenen regionalen Konzepten möglich ist. Die erfolgreiche und nachhaltige Durchführung von Wiederbelebungstrainings bedarf mindestens eines motivierten Lehrers an einer Schule.

Da Erste-Hilfe-Kurse nicht auf Wiederbelebung fokussieren und hier auch häufig trotz 9‑stündiger Dauer Zeit, ausreichend Material und Motivation für das Thema fehlen [33], ist die Kombination von Multiplikatorenschulungen für Wiederbelebungstrainings mit der Ausbildung von Ersthelfern nicht sinnvoll, auch wenn gerade Lehrer auf diesem Gebiet regelmäßige Schulungen benötigen [32]. Bekräftigt wird dies durch die Tatsache, dass in Deutschland jeder, der einen Führerschein besitzt einen Erste-Hilfe-Kurs hat und trotzdem nur sehr wenige Menschen mit Laienreanimationsmaßnahmen beginnen. Lehrer können bereits in einer Stunde [8] zu Multiplikatoren ausgebildet werden [11], um dann mit den vorhandenen Konzepten auf Schüler zugeschnittene Trainings von 45-minütiger [14] bis max. 90-minütiger Dauer effektiv und nachhaltig [34] durchzuführen. An dieser Stelle muss den Schulen nochmals kommuniziert werden, dass für die Durchführung eines Wiederbelebungstrainings weder die Teilnahme an einem Erste-Hilfe-Kurs noch der Erwerb eines Ausbilderscheins Erste Hilfe erforderlich ist. Sofern die Hilfsorganisationen über die Bundesarbeitsgemeinschaft (BAGEH) bzw. über die Landesarbeitsgemeinschaften Erste Hilfe (LAGEH) ein speziell auf Wiederbelebung fokussiertes Curriculum wie „Herzensretter“ anbieten [17], wäre die Übernahme von Schulungen für Lehrer oder sogar Schüler denkbar, nicht jedoch mit den üblichen Erste-Hilfe-Kursen. Gleiches gilt für das Programm „Retten macht Schule“ der Björn-Steiger-Stiftung [5].

Die Entwicklung eines eigenen Konzeptes wäre für die Schulen extrem aufwendig. Viele Materialen sind jedoch bereits erstellt und über Online-Plattformen von DGAI, BDA und GRC für interessierte Schulen jeglicher Schulform jederzeit und ohne Mehraufwand zugänglich. Insbesondere die interdisziplinäre Arbeitsgruppe aus Detmold entwickelt weitere Lehrmaterialen und versucht zudem, Reanimationstrainings in den Routineunterricht zu integrieren. Aus vorgenannten Gründen empfehlen die Autoren den Schulen, ein für sie geeignetes, erprobtes Konzept auszuwählen und dies mithilfe von geschulten Lehrern nachhaltig und kostengünstig zu nutzen.

Als Basis der Laienreanimation kann die alleinige Durchführung der Herzdruckmassage vermittelt werden und sinnvoll sein [21, 28], eine Erweiterung um Beatmung und den automatischen externen Defibrillator ist aufbauend möglich [29]. Die Unsicherheiten der Schüler im Regierungsbezirk Detmold, normale Atmung zu erkennen, lässt sich dadurch erklären, dass die Befragung bei diesen Schülern mehrere Monate nach den Trainings durchgeführt wurde und bis dahin Unsicherheiten wieder zugenommen hatten. Die Autoren halten es daher für sinnvoll, dass langfristig Schüler mehrmals in ihrer Laufbahn ein Reanimationstraining erhalten. Dies wird durch die signifikant besseren Ergebnisse der Schülerbefragung nach bereits in der Vergangenheit erfolgter Teilnahme an einem Training bestärkt. Vorherige Studien zeigen, dass die Kraft zur Durchführung der Herzdruckmassage ab der 7. Klasse ausreicht, die Herangehensweise jedoch bereits deutlich früher geschult werden kann [30].

Voraussetzung für eine erfolgreiche Implementierung von Wiederbelebungstrainings in Schulen ist die Finanzierung des notwendigen Materials. Aufgrund dessen ist eine Fortführung der bereits aus Spendengeldern begonnenen flächendeckenden Beschaffung von Wiederbelebungspuppen, inklusive Zubehör für alle Schulen, erforderlich. Diesbezüglich konnte sich die Beschaffung von Klassensätzen gegenüber einer Schenkung von einer Puppe an jeden Schüler durchsetzen. Ein Klassensatz bietet den Vorteil der Wiederverwendbarkeit, während eine Puppe pro Schüler eine Weitergabe der Kenntnisse im familiären Umfeld der Schüler ermöglicht [18, 25], jedoch puppenabhängig ohne Demonstrationsmöglichkeit der richtigen Drucktiefe. Hierin könnte die Unsicherheit der Schüler des Regierungsbezirks Münster in Bezug auf die Durchführung der Herzdruckmassage begründet sein.

Zusammenfassend können Menschenleben nur dann gerettet werden, wenn Wiederbelebungsmaßnahmen in Schulen in NRW durch das Ministerium für Schule und Bildung gesetzlich vorgeschrieben und koordiniert werden und von den vorhandenen Konzepten mit der notwendigen Finanzierung Gebrauch gemacht wird.

## Fazit

- Eine gesetzliche Verpflichtung und Finanzierung von Wiederbelebungstrainings nach einem Stufenkonzept ist unerlässlich für eine flächendeckende Durchführung.
- Die Schulung von Lehrern sollte gezielt auf Wiederbelebung ausgerichtet sein und kann nicht durch eine traditionelle Erste-Hilfe-Schulung ersetzt werden.
- Es muss nicht jede Schule nach dem gleichen Konzept Wiederbelebungstrainings durchführen, da ausreichend effektive Konzepte zur Verfügung stehen.
- Jede Schule sollte das am besten zu ihr passende Konzept auswählen.
- Die Errichtung einer Informationsplattform ist erforderlich, auf welcher die vorhandenen Konzepte von den Schulen abgerufen werden können.

### Limitationen

Die teilweise geringe Beteiligung bei den Befragungen kann der Corona-Pandemie geschuldet sein, da die Auswertung im Wesentlichen zu diesem Zeitpunkt stattfand.

## Caption Electronic Supplementary Material


