# Supplementary material for: Evaluation of the project for the introduction of bystander resuscitation in schools in North Rhine-Westphalia
Source: Anaesthesist. 2020 Nov 26;70(5):383–91. [Article in German] doi: 10.1007/s00101-020-00889-1 (PMC8099835; doi:10.1007/s00101-020-00889-1)
Supplement: Supplementary file 1 [file 101_2020_889_MOESM1_ESM.pdf]

Zusatzmaterial zum Beitrag "Evaluation des Projekts zur Einführung von Laienreanimation an Schulen in Nordrhein-Westfalen
Marc Felzen, Hanna Schröder, Stefan K. Beckers et al. (2020) *Der Anaesthesist* .

Beitrag und Zusatzmaterial stehen Ihnen auf www.springermedizin.de zur Verfügung. Bitte geben Sie dort den Beitragstitel in die Suche ein.

**Gesamtanzahl Schulen, Klassen + Schüler der Jahrgangsstufe 8 in NRW**		
Anzahl Schulen	2.708	
Anzahl 8. Klassen	6.540	
Anzahl Schüler Jahrgangsstufe 8	170.591	
**Einmalige Puppenbeschaffung (Vimetecsa Practiman), Haltbarkeit mindestens 5 Jahre**		
Kosten pro Puppe	43,00 €	**4.657.760,00 €**
Anzahl Puppen pro Schule	40	
**Jährliche Wartungskosten pro Puppe**		
Lungen + Mundstücke + Desinfektion	2,65 €	287.048 €
**Jährliche Schulungskosten**		
Kosten pro Lehrer	10 €	54.160 €
Anzahl Lehrer pro Schule pro Jahr	2	
**Gesamtkosten pro Jahr**		**341.208,00 €**
Kosten pro Schüler der 8. Klasse		*2,00 €*
